# Working Women in Large Towns

**Published:** 1890-11-01

**Authors:** 


### Working Women in Large Towns.

### XIV.—SOME SOURCES OF DISTRESS.

(*Continued from page* 36.)

*Foreign Competition.*—This no doubt contributes to lower the wages of English workers. In certain trades its force is very keenly felt, and in most it influences prices, and therefore wages. We must remember, however, that if it keeps down wages, it keeps down also the cost of living; and as a return to the economic fallacies of Protection cannot be recommended, all we can do is to look matters fairly in the face, and seek to hold our ground by adopting improved methods of production, and seeing, in the interests of both employers and employed, that good work is done. There are those who think that shorter hours, both among men and women, would not diminish, and actually might increase, the daily output of work. If this should be the case, it could do no harm, with regard to foreign competition, to shorten the working day; and, as regards ourselves, it would be productive of much good. But this is not a question, in our opinion, with which it would be advisable for Parliament to meddle. It should rather be left to trades unions, and given a gradual trial, lest the evils which it sought to remedy might perchance give place to more serious evils still.

*Foreign Immigration.*—East-enders will probably give you this as the cause of most of their troubles; and we cannot wonder that they have a strong feeling on this subject, when they see the shoals of foreigners in their midst, and feel themselves being continually ousted from their employment, or forced by the competition of the new-comers to take lower wages. Germany is by far the largest contributor to the foreign element in London; but as far as women are concerned, they chiefly dread the competition of foreign Jews, who come to this country knowing no trade, have the faculty of living upon almost nothing, and eagerly accept work at the very lowest wage, or even no wage at all, just to make a beginning. They are formidable rivals, these Jews, with their wonderful vitality, their soberness, prudence, and thriftiness. A few weeks will find them a step or two up the ladder; a few months may even see them "sweating" a hand or two in their working and living room. Their talent for "turning over" the minutest amount of capital has something in it almost uncanny; and this, combined with their untiring industry and their low scale of living, makes them at once the envy and the detestation of their Gentile neighbours. So strong, indeed, is the feeling against them, that some fear an outbreak of *Judenhetze* in the East-end; and thoughtful men are not wanting who, for this and for economical reasons, urge that immigration should be, at any rate partially, stopped by law. To us, however, it seems that such a plan is impossible, were it only for the fact that we must, of course, expect a *quid pro quo*, and that the closing of certain countries against us English—the most colonising and enterprising people upon earth—would probably do us more harm than our scheme would ever do us good. The influx of foreigners into our towns would seem to be, then, an infliction from which we cannot free ourselves; and this being so, we can but grin and bear it.

We may, however, commend to the attention of our readers a fact mentioned by Mr. Llewellyn Smith (in Booth's "Life and Labour of the People") which we think is not very generally known: that for the last year or so the current of Jewish immigration into this country has stopped,—or, to put it more accurately, while immigration still continues, it is balanced, and more than balanced, by emigration among these very Jews themselves. The extreme pressure of Jewish competition upon our English poor, therefore, may be looked upon as a temporary and passing evil; while with regard to the distress it has caused among poor women-workers, it is evident that the very faculties which make the Jew so dreaded by his Gentile fellow-workers must in time make him the rival of the well-to-do rather than of the poor; so that unless this stream of immigration goes on year after year (which is hardly likely), the competition of the Jews will have little permanent effect upon our lowest classes, whatever it may have in higher circles.

*The Sweating System.*—The mention of our Jewish visitors naturally leads us on to the consideration of a system in the establishment of which they have certainly had a hand. We do not mean by this that, using the word in its bad sense, they "sweat" their workers more than Gentile chamber-masters do; indeed, we may mention in this connexion that the American Commissioner of Labour expressly states that "the kindest proprietor in the world is a Jew of the better class." But the Jews certainly seem to take kindly to this special line of business, judging from the large numbers who are engaged in it; and they would not be Jews—nay, they would not be human beings, if they did not seek to make what profit they can out of it. It is, however, the system, rather than the sweater himself, that is in fault. That the sweater is a necessity where a low class of labour is employed we shall at once admit; but so many evils are connected with the sweating system (we do not say inseparably connected with it), that it would certainly seem to need examination, and perhaps control. A "terrible engine of oppression" it has been called; and such it may no doubt become. For one thing, we must point out that the contract system, which is always to be found in the sweated trades, may inflict much hardship upon the workers. It comes about in this way: The manufacturers invite tenders for a certain amount of work to be given out; the competition between the various sweaters forces them to make their tenders as low as possible; and finally a contract is accepted, so low that the workers cannot by any possibility receive a fair wage for their labour. And these contracts, be it remembered, are made without any reference to the workers. The sweater knows that if the indoor hands strike, he can find plenty of outdoor hands who will gladly undertake the job; and so the workers find themselves practically helpless. There is, in fact, no competition among employers for workers, though much among workers for work to be given out; and this, as political economists will tell us, ensures the oppression of those workers. The only remedy for this is combination, and the formation of a strong trades union, which can resist a fall in wages, and so force the manufacturers to give a better price to the contractors for the work given out. Any efforts towards such combinations among workers in these trades should be encouraged by all means in our power. Other evils connected with the sweating system will be more conveniently considered under our next heading.

*The Small Workshop System.*—The fact is that most of the evils of the sweating system are to be found among those who work, not for a large sweater, but for a small one. It is here that we find starvation-pay, long hours, filthy and overcrowded sweating-dens. The enormous multiplication of these small workshops brings with it, not only difficulty as to inspection, but economic trouble also, for these small businesses are not so profitable in proportion as are larger ones, and thus can only offer smaller wages. Again, small workshops produce irregularity of employment, for when work is slack, they can easily be closed, without any of the loss inseparable from the closing of a factory.

On the other hand, they may stand their ground under difficulties which might prove the ruin of a factory; and that they have their uses, we do not doubt. We think, however, that some controlling force is necessary, for the interests of all parties concerned. Mr. Booth suggests that all premises used for the employment of labour should be licensed; and this of itself would be a great help. Inspectors, who now have the double task of ferreting out sweating dens and then visiting them, would be entirely saved the first part of their labour; and if the system not only provided better conditions of labour, but did something to check the indefinite multiplication of sweaters, so much the better.

